# ﻿*Eriothecapaganuccii* (Bombacoideae, Malvaceae), a new endangered species from montane forests in the Atlantic Forest of Bahia, northeastern Brazil

**DOI:** 10.3897/phytokeys.243.125708

**Published:** 2024-06-27

**Authors:** Jefferson Carvalho-Sobrinho, Aline C. da Mota, Laurence J. Dorr

**Affiliations:** 1 Universidade Federal do Vale do São Francisco – UNIVASF, Colegiado de Ciências Biológicas, Petrolina, Pernambuco, 56300-990, Brazil Universidade Federal do Vale do São Francisco Petrolina Brazil; 2 Universidade Federal Rural de Pernambuco – UFRPE, Departamento de Biologia, Recife, Pernambuco, 52171-900, Brazil Universidade Federal Rural de Pernambuco Recife Brazil; 3 Universidade de Pernambuco – UPE, Instituto de Ciências Biológicas, Recife, Pernambuco, 50100-130, Brazil Universidade de Pernambuco Recife Brazil; 4 Department of Botany, MRC-166, Smithsonian Institution, P.O. Box 37012, Washington, D.C. 20013-7012, USA Smithsonian Institution Washington United States of America

**Keywords:** Bahian southern Atlantic Forest, ‘Bombacaceae’, ‘embiruçú’, endemism, plant taxonomy

## Abstract

A new species of *Eriotheca* (Bombacoideae, Malvaceae) from montane wet forests in the Atlantic Forest of Bahia, northeastern Brazil, is described and illustrated. It is known from only three populations situated between 750 m and 850 m in elevation on mountain summits and categorized as Endangered (EN) based on IUCN criteria. *Eriothecapaganuccii* is distinct from all congeners by the combination of coriaceous to strongly coriaceous leaves and remarkable few-seeded, globose to subglobose woody capsules that contain scanty kapok and the largest seeds known in the genus to date. The affinities of *E.paganuccii* to morphologically similar species as well as the importance of obtaining phenologically complete collections are discussed.

## ﻿Introduction

*Eriotheca* Schott & Endl. (Bombacoideae, Malvaceae), a genus comprised predominantly of trees, is restricted to South America. It includes 28 species of which 22 occur in Brazil mainly in Atlantic Forest and Cerrado areas (Carvalho-Sobrinho 2013; Carvalho-Sobrinho et al. 2015, 2020; Macedo et al. 2018; Yoshikawa and Duarte 2021; Duarte and Yoshikawa 2024). Three species, *E.discolor* (Kunth) A.Robyns, *E.ruizii* (K.Schum.) A.Robyns, and *E.vargasii* (Cuatrec.) A.Robyns, inhabit seasonally dry tropical forests (SDTF) reaching 3,000 m in elevation (Robyns 1963; Tropicos.org); however, no species of *Eriotheca* is known to occur in Caatinga vegetation (Duarte and Yoshikawa 2024) within the largest SDTF nucleus in South America (Pennington et al. 2009; Queiroz et al. 2017).

The current taxonomy of *Eriotheca* is grounded in studies published by Robyns (1963, 1968, 1979) and Robyns and Nilsson (1975, 1981), whose work was based mainly on analysis of herbarium collections. Robyns’ (1963) principal contribution to this genus appeared in his revision of *Bombax* L. *s.l.*, in which he accepted 20 species and four infraspecific taxa. Subsequently, the Brazilian taxa of *Eriotheca* were treated by Martins (1993) in a master’s thesis. She followed Robyns’ taxonomy but added leaf surface anatomical studies. Despite detecting some anatomical differences among taxa, she did not propose formal taxonomic changes to the genus in what remains an unpublished work.

The most recent comprehensive study on the taxonomy of Brazilian *Eriotheca* was by Duarte (2010), who in her doctoral thesis proposed taxonomic changes based on macromorphological characters and the micromorphology of scales on leaves. Her innovations were published in Duarte and Esteves (2011, 2012). Since then, several additional nomenclatural novelties from Brazil have been published, including five new species and three infraspecific taxa elevated to species rank (Duarte and Esteves 2011, 2012; Carvalho-Sobrinho 2013; Carvalho-Sobrinho et al. 2015, 2020; Macedo et al. 2018; Yoshikawa and Duarte 2021).

Historically, the taxonomy of *Eriotheca* has been challenging for several reasons: i) Individual trees often reach 40 m in height and making herbarium specimens requires climbing equipment or tree climbers; ii) Wet forest species often have supra-annual flowering; iii) Leaf morphology is highly variable within species and within individuals (even on the same branch) as well as between vegetative and reproductive branches; iv) Reproductive branches are often leafless resulting in herbarium collections consisting of separate branches and often including much larger leaves from young individuals; v) A lack of standardization in the collection and description of leaves, including proximal and distal leaflets; vi) Fruit and seed are seldom included on herbarium sheets and rarely linked to well-curated carpological collections; vii) Type material of *Eriotheca* species is typically incomplete phenologically and often comprised of poorly preserved reproductive and vegetative parts with loose bits such as fragments of leaflets, flowers, capsules, and seeds.

Except for a few species (see e.g., Robyns 1968; Carvalho-Sobrinho et al. 2015, 2020; Yoshikawa and Duarte 2021), protologues of *Eriotheca* characteristically lack either illustrations or descriptions of fruit and seed (or both). In Robyns’ (1963) revision, seven taxa of *Eriotheca* were described without fruit and seed descriptions. Despite a doctoral thesis on the Brazilian species of *Eriotheca* (Duarte 2010), the morphology of fruit and seed of many Brazilian species is still unknown. This hampers a thorough understanding of the taxonomy of the genus. Nonetheless, taxonomic decisions including the synonymizing of taxa have been undertaken without a complete knowledge of the morphology of the fruit and seed of the taxa involved (e.g., Duarte 2010; Duarte and Esteves 2012) and new species have been described based on phenologically incomplete material (e.g., Fernández-Alonso 2003; Duarte and Esteves 2011; Macedo et al. 2018).

Thus, long-term monitoring of populations of Neotropical Bombacoideae in the field has been undertaken, especially in northeastern Brazil, and has produced phenologically complete herbarium collections including fruit and seed (see e.g., Carvalho-Sobrinho 2013; Carvalho-Sobrinho and Queiroz 2008, 2010; Carvalho-Sobrinho et al. 2012, 2013a, b, 2014, 2015, 2020, 2021; Carvalho-Sobrinho and Dorr 2017, 2020).

The Atlantic Forest of northeastern Brazil, particularly the southern Atlantic Forest of Bahia, harbors remarkable levels of plant richness and endemism (Amorim et al. 2005, 2008, 2009; Thomas et al. 2008; Amorim and Matos 2009). Recent field efforts in this region have revealed specimens of *Eriotheca* that are noteworthy due to their capsules containing scanty kapok and relatively few seeds that are much larger than those occurring in other species of the genus. These specimens have flowers typical of *Eriotheca*, which differ morphologically from the phylogenetically related genus *Pachira* Aubl. *Eriotheca* flowers are consistently smaller with reniform (vs. oblong-linear) anthers and they lack (vs. possess) phalanges on the androecium (Carvalho-Sobrinho et al. 2016).

Careful study of these collections has led to the recognition of a new species, which is described and illustrated here. None of the collections has been cited previously in the literature including in the last taxonomic treatment of *Eriotheca* for Brazil by Duarte (2010). Notes on the distribution and phenology of this new species, along with comments on morphologically similar species, and an assessment of the conservation status of this novelty, are provided. Also highlighted is the importance of having phenologically complete collections for studies in this and closely related genera.

## ﻿Material and methods

This study was based on examination of herbarium collections, digital images of specimens, and field observations. Specimens were studied by visits to or loans from the following herbaria: ALCB, ASE, BAH, CEPEC, F, G, HRB, HUEFS, IPA, K, M, MBM, MO, NY, P, PEUFR, R, RB, SP, SPF, UESC, UFPB, UFPE, UFRN, and US (acronyms according to Thiers 2021). A comprehensive analysis of images of herbarium specimens was studied through the following websites: INCT – Herbário Virtual da Flora e dos Fungos (http://inct.splink.org.br/), JSTOR Global Plants (https://plants.jstor.org/), and Reflora Virtual Herbarium (https://reflora.jbrj.gov.br). Descriptions and measurements were based on dry herbarium specimens unless otherwise clearly stated. The distribution map was prepared using the free and open source QGIS software. A preliminary extinction risk assessment of the new species was made based on the IUCN criterion B (IUCN 2024). Georeferenced specimen data were imported into GeoCAT (Bachman et al. 2011) to estimate the extent of occurrence (EOO) and the area of occupancy (AOO) using 2 × 2 km grid cells.

## ﻿Taxonomic treatment

### ﻿ Eriotheca paganuccii

Taxon classificationPlantaeMalvalesMalvaceae

Carv.-Sobr., A.C.Mota & Dorr sp. nov.

3903F0E3-EE36-5156-8987-069395F03015

urn:lsid:ipni.org:names:77344361-1

**Figure 1.**
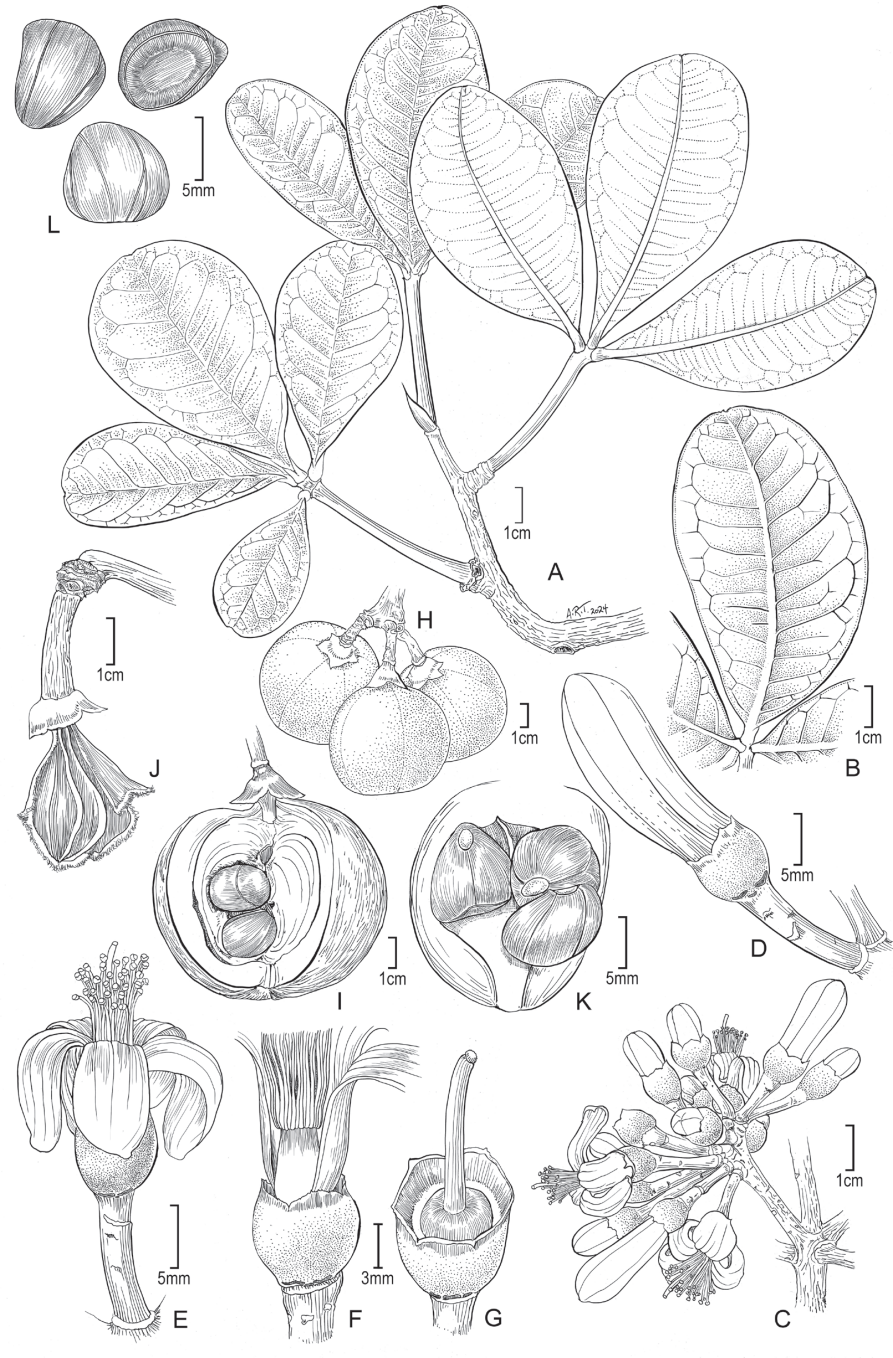
*Eriothecapaganuccii***A** vegetative branch from the top of the tree canopy **B** details of a leaflet also from the top of the tree canopy **C** leafless branch with umbelliform cymes **D** flower bud (note the glands on receptacle) **E** flower at anthesis (note the unilaterally apiculate petals) **F** staminal tube (detached from the receptacle and slightly pulled up) **G** gynoecium **H** globose woody capsules before dehiscence **I** capsule with one valve detached showing scanty kapok and large seeds **J** dehisced fruit with marcescent calyx, exposing the columella and remaining kapok after seed dispersal **K** seeds relative to a fruit valve **L** angulate, striated seeds; each seed with two plane and one concave sides. **A, B, I–L** drawn from *J.G. Carvalho-Sobrinho 4040*, **C–G** drawn from *J.G. Carvalho-Sobrinho & A.C. Mota 4022*.

**Figure 2.**
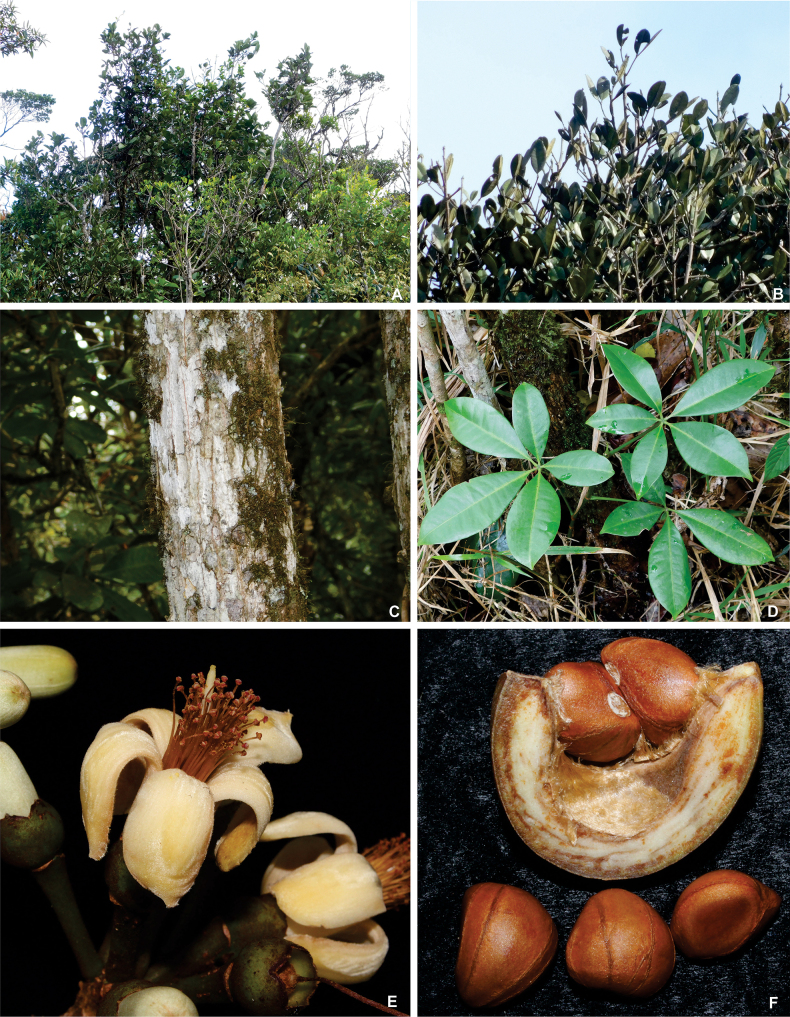
Habitat and morphological aspects of *Eriothecapaganuccii***A** crown of *E.paganuccii* individuals in the canopy **B** detail of leaves at the top of the tree canopy showing smaller, more rigid leaves that are comprised of 1–2 leaflets oriented upward **C** bark of *E.paganuccii***D** leaves with less exposure to the sun that have 5 leaflets, which are larger, less rigid, and patent (not oriented upward) unlike those in the canopy **E** flower at anthesis **F** seeds relative to a fruit valve and scanty kapok.

**Figure 3.**
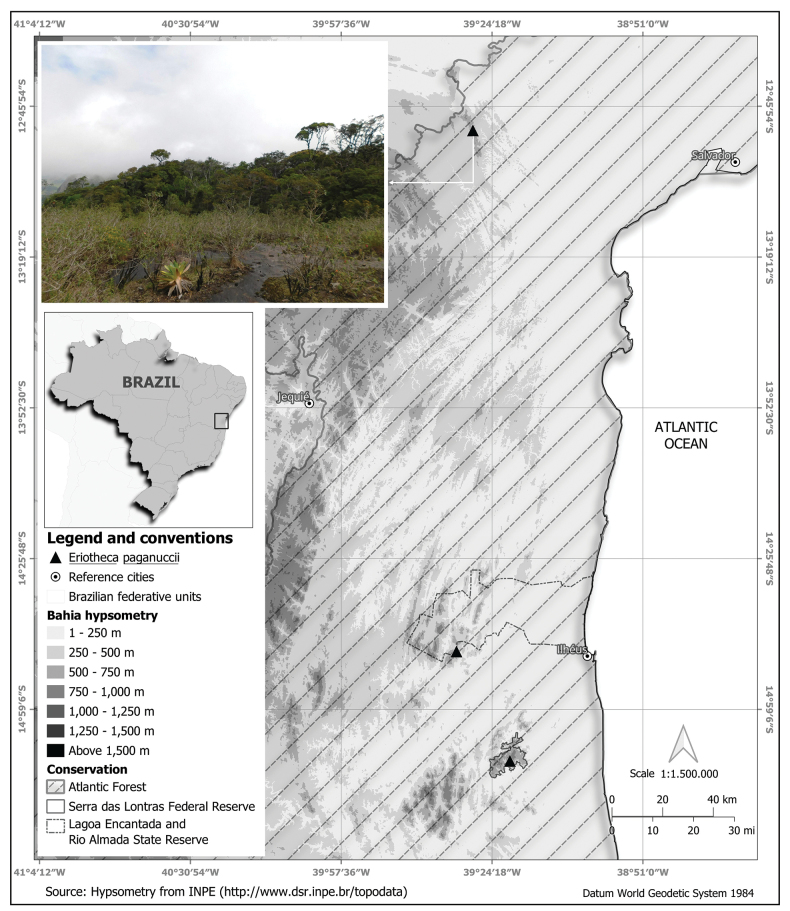
Distribution map of *Eriothecapaganuccii* and type-locality with montane wet forest between 750 m and 850 m in elevation near granitic-gneissic rock outcrops on the summit of Serra da Jiboia in the Atlantic Forest of Bahia, northeastern Brazil.

Figs 1 , 2 , 3 , Table 1

#### Diagnosis.

Similar to *Eriothecaobcordata* A.Robyns due to its absence of buttresses, obcordate leaflets, oblong to oblanceolate flower buds, oblanceolate petals, and stamens c. 80 in number, but differing in its caducous (vs. often persistent) bracteoles, larger calyces (7 × 7–9 mm vs. 5–5 mm), globose to subglobose (vs. obovoid) capsules, scanty (vs. abundant) kapok, seed number (c. 10 vs. numerous) per fruit, seed size (10–19 mm vs. 5–7 mm) long, and marcescent, lignified calyces that often split into patent lobes (in herbarium collections, at least).

#### Type.

Brazil. Bahia: Castro Alves, Serra da Jiboia (=Serra da Pioneira), Mata higrófila, 12°51'11"S, 39°28'19"W, 22 Dec 1992 (lf, fr), *L.P. Queiroz* & *T.S.N. Sena 3008* (Holotype: HUEFS barcode 000132176! Isotype: SP barcode 057771!).

#### Description.

Trees 2.5–5(–8) m tall, lacking buttresses; branches glabrous, often lenticellate; fertile branches often suberized. Terminal bud often persistent at branch apex, 11–15 mm long, acute at apex of mature branches, often curved at apex of younger branches. Leaves palmately compound; petiole doubly-pulvinate, (15–)40–65 × 2–3 mm, cylindric, swollen at base when fresh; petiolule greatly reduced; leaflets 3–5 (often 1–2-foliolate on canopy branches), coriaceous to strongly coriaceous, glabrous on both surfaces, discolorous, margin entire, revolute, the adaxial surface covered by dense indumentum of scales with irregular outline, the abaxial surface light green when fresh, the midrib yellowish adaxially, strongly prominent abaxially, secondary veins 10–13, prominent on abaxial surface, impressed on adaxial surface, the tertiary veins impressed on abaxial surface; leaflets often naviculate when young, distal leaflets 48–190 × 35–88 mm, length-to-width ratio 1.4–2.7, obovate, obcordate, widely-elliptic, or rarely elliptic, apex obtuse, truncate to retuse, base acute, decurrent or obtuse, rarely falcate; proximal leaflets 24–161 × 15–79 mm, length-to-width ratio 1.4–2, elliptic to widely-elliptic or elliptic-obovate, apex apiculate, base acute, decurrent, or obtuse. Inflorescences 1–8-flowered umbelliform cymes, borne on terminal, often suberized, leafless branches; pedicels 11–25 × 2.5(–4 at apex) mm, with sparse scales, green to blackish-green when fresh, the bracteoles ovate, caducous. Flower buds oblongoid when young to narrowly obovoid just before anthesis, receptacle c. 1–2 mm long, glandular, rarely eglandular; calyx 7–8 × 7–8 (–10 mm when compressed on herbarium sheets), glabrous except for sparse scales, urceolate when fresh, cupuliform to campanulate on herbarium sheets, apex inconspicuously crenulate to shortly 5-lobed, the lobes irregularly-shaped to triangular, marcescent in fruit, the outer surface green to blackish-green covered with sparse ferruginous indumentum; petals 23–26 (31–32 when fresh) × 4 mm at the base to 8 mm at the apex, cream-colored to pale yellow on both faces when fresh, greyish-brown when dried, reflexed by the distal length, oblanceolate, unilaterally apiculate and curved, tomentose on both faces, internally with longitudinal lines of longer trichomes on one longitudinal half (sericeous); stamens c. 80, reddish-brown when fresh, the staminal tube 7 × 4 mm, slightly enlarged at ovary height, slightly constricted at apex, producing free filaments 20 mm long; ovary globose, densely ferruginous indument at the distal half, style white, glabrous, stigma light-green. Loc­ulicidal capsules woody, globose to subglobose, 35–50 mm long (–70 mm, when fresh), length-to-width ratio 0.8–0.9, externally ferruginous when young, the indument caducous at maturity, the kapok scanty, golden, the marcescent, lignified calyces often splitting into patent lobes on herbarium sheets. Seeds c. 10 per fruit, (11–)14(–19) × (10–)11(–14) × (9–)10(–12) mm (L×W×H), brown, glabrous, angulate, three-sided (two plane and one concave), 5-striate, three striae often coinciding with seed corners, two striae on dorsal (opposite the hilum) side, striae the same color as the testa.

#### Phenology.

*Eriothecapaganuccii* was collected in flower in September (very young flower buds) and December, and in fruit in October (very young fruits), December to February.

#### Distribution and habitat.

*Eriothecapaganuccii* is endemic to montane wet forests found between 750 m and 850 m in elevation near granitic-gneissic rock outcrops on summits of mountains in the Atlantic Forest of Bahia, northeastern Brazil (Fig. 3). Remnants of Montane Forest in the Atlantic Forest of Bahia are known for their outstanding diversity and include several endemic angiosperm taxa in *Bertolonia* Raddi, *Dichorisandra* J.C.Mikan, *Macrocarpaea* (Griseb.) Gilg, and *Quesnelia* Gaudich. (Coelho and Amorim 2014). In particular, the Serra da Jiboia is also the type locality of several species of algae (e.g., *Diplocladiellacornitumida* F.R.Barbosa et al.), fungi (e.g., *Anteagloniumbrasiliense* D.A.C.Almeida et al.; *Diplococciumvariegatum* S.S.Silva et al.; *Thozetellasubmersa* F.R.Barbosa & Gusmão), and angiosperms (e.g., *Heteropterysjardimii* Amorim, *Marantavillosovaginata* N.Luna & E.M.Pessoa, and *Passiflorajiboiaensis* M.A.Milward de Azevedo). These latter three species are endemic to the state of Bahia. Additional angiosperms endemic to Bahia that occur in Serra da Jiboia include *Eugeniaaltissima* Sobral & Faria (Endangered – EN category), *Ingaconchifolia* L.P.Queiroz (Endangered – EN category), *Ormosiatimboensis* D.B.O.S.Cardoso et al. (Critically Endangered – CR category), and *Sennabahiensis* A.G.Lima & V.C.Souza (Critically Endangered – CR category), all of which are threatened with extinction according to CNCFlora (2024).

#### Conservation status.

*Eriothecapaganuccii* is known only from seven collections made at three different localities (IUCN B2a criterion), which qualifies it for the Endangered (EN) category. The extent of occurrence (EOO) of this species has been calculated to be 1,738 km^2^, which qualifies the species for the Endangered (EN) category, and the area of occupancy (AOO) was estimated to be 12 km^2^, which also qualifies it for the Endangered (EN) category according to B1 criterion (IUCN 2024). Two of the three known populations of *E.paganuccii* inhabit legally protected Reserves: one population occurs within a federal level Reserve “Parque Nacional Serra das Lontras” and one population within the state level protected “Área de Proteção Ambiental Lagoa Encantada”. However, the Atlantic Forest habitat has been lost at an accelerated rate due to anthropogenic pressures (B2b criterion). Therefore, due to the very restricted extent of the habitat of this species (montane wet forests on mountain summits), the rapid rate of deforestation of the surrounding Atlantic Forest, and the small AOO (12 km^2^) and EOO (1,738 km^2^) of *E.paganuccii*, we consider this species to be Endangered belonging to the EN B1ab(i,iii), B2ab(ii,iii) category based on available data and IUCN criteria (IUCN 2024).

#### Etymology.

The specific epithet honors Luciano Paganucci de Queiroz, a distinguished Brazilian taxonomist who was the first to collect this new species and one of the first to promote and undertake floristic efforts on Serra da Jiboia, Bahia, its type-locality.

#### Additional specimens examined.

Brazil. Bahia: **Arataca**, RPPN do IESB, Serra do Peito de Moça, Rod. Arataca/Una, entrada 9,5 km da cidade, mais 8,9 km da entrada, topo da serra, 15°10'27"S, 39°20'22"W, 20 Dec 2008 (lf, fr), *A.B. Jardim et al. 168* (CEPEC 127300!, RB barcode 00734410). **Barro Preto**, estrada de terra para Pedra Lascada, cume da serra, 14°46'17"S, 39°32'10"W, 841 m alt., 13 Feb 2011 (lf), *J.G. Carvalho-Sobrinho & A.C. Mota 2902* (HUEFS barcode 000132932!). **Santa Terezinha**, Serra da Jiboia, c. 4 km de Pedra Branca, Mata Higrófila, 12°51'11"S, 39°28'19"W, 27 Sept 2000 (lf, fl buds), *L.P. Queiroz 6370* (CESJ 44810, HUEFS barcode 000132068!, SP barcode 057770!). Same municipality, 14,5 km na rod. Elísio Medrado/Santa Terezinha, Torre da Embratel, c. 7 km do distrito de Pedra Branca, Serra da Jiboia, Campos de Altitude, 12°51'13"S, 39°28'33"W, 750 m alt., 24 Feb 2000 (lf, fr), *J.G. Jardim et al. 2808* (ALCB barcode 062988, BAH 5228, CEPEC 88453!, HUEFS barcode 000138269!, NY barcode 00566377!, RB barcode 00778244!, SPF barcode 00161825, UESC 7099, UFRN 13793!); same municipality, Serra da Jiboia, Morro da Pioneira, cume, 12°51'18"S, 39°28'33"W, 817 m alt., 28 Oct 2023 (lf, fl), *J.G. Carvalho-Sobrinho & A.C. Mota 4022* (CEPEC, HUEFS, IPA, RB, SP, SPF); same locality, 28 Oct 2023 (lf, very young fruits), *J.G. Carvalho-Sobrinho & A.C. Mota 4023* (HUEFS, IPA); same locality, 25 Jan 2024 (lf, fr), *J.G. Carvalho-Sobrinho 4038* (HUEFS barcode 000100270218!); same locality, 25 Jan 2024 (lf), *J.G. Carvalho-Sobrinho 4039* (HUEFS barcode 000100270219!); same locality, 25 Jan 2024 (lf, fr), *J.G. Carvalho-Sobrinho 4040* (HUEFS barcode 000100270220!).

## ﻿Discussion

### ﻿Taxonomic affinities of *Eriothecapaganuccii*

*Eriothecapaganuccii* is characterized by flowering branches with caducous leaves, often suberized, leaflets coriaceous to strongly coriaceous (chartaceous when young), leaves 3(–5)-foliolate, and fruit globose to subglobose that are 35–50 mm long (–70 mm when fresh). The woody capsules with scanty kapok enclosing few seeds (c. 10 per capsule) are unique characteristics among species of *Eriotheca*. Moreover, the seeds that are 11–19 mm long are the largest in the genus encountered to date.

Among species found in Bahia, *Eriothecapaganuccii* is similar to *E.ob­cordata* due to its absence of buttresses, obcordate leaflets, oblanceolate petals, stamens c. 80 in number, and staminal tube length-to-width ratio of c. 1.5, but it differs by the presence (vs. absence) of glands on the receptacle, caducous (vs. often persistent) bracteoles, larger (7 × 7–9 mm vs. 5–5 mm) calyces, and globose to subglobose (vs. obovate) capsules (Table 1).

**Table 1.** Comparison of *Eriothecapaganuccii* to morphologically similar species in the state of Bahia, northeastern Brazil.

Trait	*E.alversonii*	*E.macrophylla*	*E.obcordata*	*E.paganuccii*
Buttresses	present	present	absent	absent
Outline of scales on leaves	unknown	regular outline (Duarte 2010)	regular outline (Duarte 2010)	irregular outline (Duarte 2010)
Glands on receptacle	absent	present	absent	present, rarely absent
Bracteoles	caducous	caducous	often persistent	caducous
Flower bud shape	oblong	widely-elliptic	oblong	oblanceolate
Calyx dimensions (mm)	3–4 × 3–5	5–6 × 7–9	5 × 5	7 × 7–9
Calyx apex	truncate to crenulate	crenate to crenulate	truncate	inconspicuously to shortly 5-lobed
Petal dimensions (mm)	15–23 × 3–6	30 × 10–12	21–25 × 7	23–26 × 4–6
Number of stamens	c. 70	c. 140	c. 80	c. 80
Staminal tube length (mm)	4–5	4 × 3.5	5–6 × 1.5–1.7	7 × 4
Fruit kapok	abundant	abundant	abundant	scanty
Fruit length (mm)	15–21	38–60	28–60	35–50(–70, when fresh)
Fruit shape	globose to subglobose	obovoid to subglobose	obovoid	globose to subglobose
Seed length (mm)	c. 5	c. 10	c. 5	(11–)14(–19)
Seed number per fruit	numerous	numerous	numerous	c. 10
Flowering period	July to September	October to December	November to April	September (very young flower buds); December
Fruiting period	August to October, December to February	December to February	February	October (very young fruits); December to February

*Eriothecapaganuccii* has been misidentified in herbaria as *E.macrophylla* (K.Schum.) A.Robyns. Both species share inflorescences borne on branches that are often leafless and modified as brachyblasts, caducous bracteoles, and glands on the receptacles (Table 1). However, *E.paganuccii* can be readily distinguished from *E.macrophylla* by its oblanceolate (vs. widely-elliptic) flower buds, short (c. 1 mm vs. 2–5 mm long) receptacles, oblanceolate (vs. obovate) petals that are 4–8 mm (vs. 10–12 mm) wide and with a length-to-width ratio of 5 (vs. 3), fewer (c. 80 vs. c. 140) stamens, and staminal tube length-to-width ratio of 1.5–2 (vs. up to 1.2).

*Eriothecapaganuccii* differs from the two aforementioned species also by the presence of scales with an irregular outline on leaves (vs. scales with regular outline according to Duarte and Esteves 2011). Moreover, *E.paganuccii* is endemic to montane wet forests near 800 m in elevation whereas *E.macrophylla* and *E.obcordata* inhabit mainly low altitudinal areas in the Atlantic coast.

*Eriothecapaganuccii* is also morphologically similar to *E.alversonii* because the two species may present 3-foliolate leaves with obovate leaflets, oblong flower buds, oblanceolate petals, and globose to subglobose capsules. However, *E.paganuccii* can be distinguished by its leafless (vs. leafy) flowering branches, very coriaceous (vs. chartaceous) leaflets, glabrous (vs. with sparse, darkish indumentum) pedicels, receptacles and calyces, larger calyces (7 × 7–9 mm vs. 3–4 × 3–5 mm), larger capsules (35–50 mm vs. 15–21 mm), scanty (vs. abundant) kapok, and seeds c. 14 mm (vs. c. 5 mm) long.

*Eriothecapaganuccii* is morphologically similar to *E.pentaphylla* (Vell.) A.Robyns, a species endemic to the southeastern Atlantic Forest in the states of Rio de Janeiro and São Paulo (Duarte and Yoshikawa 2024), both species with relatively large seeds, 7–14 mm vs. (11–)14–18 mm long, respectively. Additionally, herbarium specimens of these two species often present marcescent, lignified calyces that split into patent lobes. However, *E.paganuccii* differs from *E.pentaphylla* in its smaller calyces (7 × 7–9 mm vs. 8–12 × 13 mm) that are inconspicuously to shortly 5-lobed (vs. distinctly lobed), narrower petals (4–8 mm vs. 11–15 mm wide), white (vs. often pinkish) filaments, and globose to subglobose (vs. obovoid) capsules that are c. 35–50(–70) mm long (vs. 80–100 × 45 mm). Furthermore, *E.paganuccii* flowers from November to December and fruits from December to February whereas *E.pentaphylla* flowers from April to July and fruits from August to November.

### ﻿Leaf morphology variation and the importance of phenologically complete collections

Typically, species of *Eriotheca* and other Bombacoideae present distal and proximal leaflets that are distinct in shape and size. This can be observed in *E.paganuccii*, in which distal leaflets can be longer (length-to-width ratio of 1.4–2.7) than proximal ones (length-to-width ratio of 1.4–2). *Eriothecapaganuccii* also presents great variation in its leaves according to their position on the tree (Fig. 2D, F). During fieldwork, we observed that leaves on branches at the top of the tree canopy are generally distinct from leaves on branches with less exposure to the sun: they are smaller and consist of fewer (often one or two), more rigid leaflets that are oriented upwards (Fig. 2D). This high plasticity in leaf morphology has also been observed on herbarium specimens of other *Eriotheca* from the Atlantic Forest and can make taxonomic identification difficult, especially for taxa originally described based on few or only one specimen, as is frequently observed in the genus.

This idiosyncratic way in which Bombacoideae herbarium specimens are made (Carvalho-Sobrinho and Queiroz 2008, 2010; Carvalho-Sobrinho et al. 2013a, b, 2014) also highlights the taxonomic importance of the morphology of reproductive organs especially fruit and seed as demonstrated by recent studies (Carvalho-Sobrinho et al. 2020; Yoshikawa and Duarte 2021). Regrettably, morphological descriptions of both fruit and seed of ten species of *Eriotheca* have not yet been published including the following six species known from the Atlantic Forest: *E.bahiensis* M.C.Duarte & G.L.Esteves, *E.crenulaticalyx* A.Robyns, *E.dolichopoda* A.Robyns, *E.longipes* (A.Robyns) M.C.Duarte & G.L.Esteves, *E.macrophylla*, and *E.platyandra* A.Robyns.

As a consequence, a number of herbarium specimens of *Eriotheca* represented by only fruit continue to have inaccurate taxonomic identifications. *Eriothecapaganuccii* can serve to illustrate this situation: it was first collected in 1992 with only fruits, and again with only fruits in 2000 and 2008. These incomplete specimens were tentatively identified as *E.globosa*, *E.macrophylla*, and *E.obcordata*. The morphology of flowers, however, collected in October 2023 allowed *E.paganuccii* to be clearly distinguished from these *Eriotheca* species as demonstrated above.

An additional example of the impact of phenologically complete collections for plant taxonomy and conservation can been observed in *Eriotheca platy­andra* (Robyns 1963) whose identity remains doubtful to the point of its being overlooked in the treatment of the genus for “Flora e Funga do Brasil” (Duarte and Yoshikawa 2024).

Here we provide further evidence for the importance of fruit and seed characters to the taxonomy of *Eriotheca* especially for circumscribing species from Atlantic Forest that present fairly conservative floral morphologic traits and represent the major taxonomic challenge remaining in the genus (Carvalho-Sobrinho, pers. observ.). The greater morphological and taxonomic diversity of *Eriotheca* observed in the Atlantic Forest demands further efforts toward resolving the taxonomy of the genus and eventually may be linked to complex dynamics of speciation, as observed in *Eriotheca* from the Brazilian Cerrado. The existence of polyploid populations associated with distinct fruit and seed traits has been linked to reproductive strategies in Cerrado lineages. Such findings have revealed the existence of a species complex (Mendes-Rodrigues et al. 2019; Marinho et al. 2020) and can help us understand the origins of the high morphological variability observed in *Eriotheca* species of the Cerrado.

Cytogenetic and cytomolecular data also have revealed noteworthy biogeographic and species richness patterns in *Eriotheca* and allied genera that shed light on its evolutionary history as well as on the relationship of *Eriotheca* with the closely related genus *Pachira* (Costa et al. 2017). Furthermore, these data have been analyzed in a phylogenetic framework and indicated neopolyploidy may be involved in speciation of *Eriotheca* lineages from the Atlantic Forest (Costa et al. 2017).

Therefore, in order to improve the taxonomy of *Eriotheca* and facilitate the production of cytogenetic and cytomolecular data, it is critical to obtain phenologically complete herbarium collections including fruit and seed of each species and, whenever possible, each population. Such efforts will enhance the systematics of *Eriotheca* allowing the evaluation of the evolutionary processes underpinning the diversity in this group especially in Atlantic Forest lineages that are threatened with extinction.

## Supplementary Material

XML Treatment for Eriotheca paganuccii

## ﻿Additional information

### Author ORCIDs

Jefferson Carvalho-Sobrinho https://orcid.org/0000-0003-3605-0707

Aline C. da Mota https://orcid.org/0000-0002-8440-1420

Laurence J. Dorr https://orcid.org/0000-0001-7157-363X

### Data availability

All of the data that support the findings of this study are available in the main text.

## ﻿Appendix 1

**Herbarium specimens examined of two *Eriotheca* species in Bahia morphologically similar to *E.paganuccii***

*Eriothecamacrophylla* (K.Schum.) A.Robyns

**Brazil. Bahia.** Ilhéus, on road to Vila Brasil, 10 km West of junction with BA001, the junction c. 40 km south of Ilhéus, just north of the Rio Acuípe, 15°06'S, 39°04'W, 10 May 1993 (lf, fl), *W. Thomas et al. 9843* (CEPEC, NY barcode 00998006, SP, US barcode 01220017). Itacaré, entre a Praia do Farol e a Praia da Ribeira, 14 Dec 1992 (lf, fl), *A. Amorim et al. 951* (CEPEC, HUEFS barcode 000138305, NY barcode 00402470, US barcode 01226959). Itapebi, Faz. Dois Irmãos, Rodovia para Potiraguá, 10 Nov 1970 (lf, fl), *R.S. Pinheiro* & *T.S. Santos 419* (CEPEC). Jequié, Fazenda Brejo Novo, a 10,5 km da Av. Otávio Mangabeira entrado pela Exupério Miranda no Bairro do Mandacaru, 13°56'53.6"S, 40°06'42"W, 716 m, 08 Dec 2004 (st), *G.E.L. Macedo & J.L. Paixão 1499* (HUEFS). Porto Seguro, parte sul entre os municípios de Ajuda e Porto Seguro, 08 Nov 1963 (lf, buds), *A.P. Duarte 7999* (RB barcode 00059485, SP barcode 057769). Santa Cruz de Cabrália, Res. Bio. Pau-Brasil, 11 Dec 1971 (lf, fl), *A. Eupunino 94* (CEPEC). **Espírito Santo.** Conceição da Barra, Área 157 da Aracruz Celulose S.A., 28 Oct 1993 (lf, buds) *O.J. Pereira et al. 5163* (VIES). Linhares, Reserva Natural da CVRD, estrada Flamengo, km 08, 03 Dec 2004 (lf, buds), *D.A. Folli 4999* (CVRD); *ibidem*, Reserva Natural da CVRD, estrada Flamengo, km 07, próximo ao pátio, 14 Jan 1994 (lf, fr), *D.A. Folli 2170* (CVRD). **Pernambuco.** Brejo da Madre de Deus, Mata do Bituri, Serra do Prata, próximo do mirante, 08°12'27"S, 36°23'32"W, 920–1030 m, *L.M. Nascimento 195* (HUEFS barcode 000132032, PEUFR, RB barcode 00059255).

*Eriothecaobcordata* A.Robyns

**Brazil. Bahia.** Buerarema, estrada São José da Vitória-Buerarema, ramal à direita, estrada de acesso a Pedra Branca, 15°05'S, 39°19'W, 15 Oct 2003 (lf, fl), *P.Fiaschi et al. 1709* (CEPEC, HUEFS barcode 000138548, MO barcode 2256429, NY barcode 01092733, SPF barcode 162815). Entre Rios, Fazenda Mangueira, 11°53'8"S, 37°57'13"W, 90–100 m, 26 Feb 2005 (lf, fr), *J.G. Carvalho-Sobrinho 365* (HUEFS barcode 000138548). Entre Rios, Fazenda Mangueira, 27 Feb 2010 (lf, fr), *J.G. Carvalho-Sobrinho et al. 2660* (HUEFS barcode 000138053). Entre Rios, Fazenda Mangueira, 28 Feb 2010 (lf, fl, fr), *J.G. Carvalho-Sobrinho et al. 2711* (HUEFS barcode 000138584). Ilhéus, Ribeirão da Fortuna, 23 June 1944 (lf, fl), *H.P. Velloso* (HUEFS barcode 000138053).
